# Prenatal Alcohol Exposure and the Risk of Depression in Offspring: a Meta-Analysis

**DOI:** 10.1155/2022/5458611

**Published:** 2022-04-30

**Authors:** Xiaoming Zhang, Yanru Liu, Jing Li, Binbin Li, Xingjie Yang, Qi Sun, Jingyi Yan, Zhiren Wang, Huaqing Liu

**Affiliations:** ^1^Department of Psychiatry, Beijing Huilongguan Hospital, Beijing 100096, China; ^2^Chengde Medical University, Chengde 067000, Hebei, China

## Abstract

**Background:**

Prenatal alcohol exposure (PAE) has been related to poor consequences of mental health in offspring. However, it remains unknown whether maternal alcohol drinking during pregnancy is associated with depression in the offspring.

**Methods:**

A meta-analysis was performed accordingly. Relevant observational studies were identified from Medline, Embase, and Web of Science databases. A fixed-effect or a random-effect model was selected dependending on the between-study heterogeneity.

**Results:**

Eight cohort studies were included. The heterogeneity was not significant (*I*^2^ = 14%). A meta-analysis with a fixed-effect model showed that PAE was associated with a higher risk of depression in offspring (odds ratio (OR): 2.28, 95% confidence interval (CI): 1.61 to 3.25, *p* < 0.001). Subgroup analysis showed that moderate (OR: 1.74, 95% CI: 1.22 to 2.49, *p*=0.002, *I*^2^ = 0%) or heavy (OR: 2.41, 95% CI: 1.55 to 3.73, *p* < 0.001, *I*^2^ = 0%) maternal alcohol drinking in pregnancy was associated with depression in offspring, but not for those with low maternal alcohol drinking (OR: 1.18, 95% CI: 0.97 to 1.44, *p*=0.10, *I*^2^ = 0%). Further subgroup analyses according to study design, timing of PAE evaluation, age at depression diagnosis, and quality scores showed consistent results. Univariate metaregression showed a dose-response association between PAE and offspring depression (coefficient: 0.073, 95% CI: 0.019 to 0.127, *p*=0.014).

**Conclusions:**

Current evidence suggests that PAE may be a risk factor of depression in offspring.

## 1. Introduction

Depression is a common mental health problem not only in adults but also in children and adolescents [1, 2]. It has been indicated in previous studies that depression in children and adolescents is often underdiagnosed but has similar clinical features and adverse influences to depression in adults [3, 4]. Depression in children and adolescents is also characterized by mood disturbances, sadness, irritability, guilt, and loss of interest to almost all activities, which could lead to poor social and academic performance, substance use disorders, self-harm, or even suicide [5–8]. Accordingly, the identification of risk factors for depression in children and adolescents is critical for the development of preventative strategies for the affective disorder. Alcohol consumption is prevalent in pregnant women, with an estimated global prevalence of 10% [9]. It has been suggested that prenatal alcohol exposure (PAE) may be related to a variety of adverse maternal and neonatal outcomes in previous studies, including miscarriage, small-for-gestational age, and preterm delivery [10–12]. Besides, accumulating evidence suggests that PAE may also be related to with a higher risk of depression in offspring, probably via dysregulation of the hypothalamic-pituitary-adrenal (HPA) axis [13–15]. However, previous observational studies evaluating the association between PAE and depression in offspring showed inconsistent results [16–23]. Some studies supported a significant association between PAE and offspring depression [16, 17, 19, 22, 23], while others did not [18, 20, 21]. Accordingly, we performed a meta-analysis of cohort studies to comprehensively evaluate the relationship between PAE and the risk of offspring depression.

## 2. Methods

We followed the Preferred Reporting Items for Systematic Reviews and Meta-Analyses (PRISMA 2020) [24, 25] and Cochrane's Handbook [26] guidelines during the design, performing, and presenting of the meta-analysis.

### 2.1. Search of Electronic Databases

We identified studies by a systematic search of Medline, Embase, and Web of Science electronic databases using the following terms: (1) “maternal” OR “prenatal” OR “pregnant” OR “pregnancy” OR “periconception” OR “gestation” OR “antenatal” OR “perinatal;” (2) “alcohol” OR “alcoholic” OR “ethanol;” and (3) “depression” OR “depressive” OR “affective disorder”. Only studies in English were selected. An additional manual check-up for the reference lists of relevant original and review articles was also performed as a supplement. The last literature search was conducted on October 10, 2021.

### 2.2. Selection of Eligible Studies

The PICOS criteria were used for study inclusion.

1. P (Participants): pregnant women and their offspring
2. I (Intervention/exposure): children or adolescent with PAE
3. C (Control/comparator): children or adolescent without PAE
4. O (Outcome): depression in offspring, relative risks, and their 95% confidence interval (CI) reported
5. S (Study design): cohort studies: the diagnosis of depression was consistent with the methods applied in the original articles. Reviews, preclinical studies, studies that did not evaluate maternal alcohol consumption during pregnancy, or studies that did not report the outcome of depression in offspring were excluded.

### 2.3. Extraction of Data and Evaluation of Study Quality

Two of the authors independently conducted electronic database search, extraction of study data, and assessment of study quality according to the inclusion criteria previously described. If there were discrepancies, discussion with the corresponding author was indicated to resolve them. The extracted data included the following: (1) name of the first author, year of the publication, study design, and country of the study; (2) number of children included, timing of PAE assessment, and methods for the validation of PAE; (3) categories used for quantitative evaluation of PAE in each study; (4) methods for the diagnosis of depression in offspring, and average age of children/adolescents at the diagnosis of depression; and (5) variables adjusted in the multivariate analyses for the association between PAE and depression in offspring. The Newcastle–Ottawa Scale (NOS) [27] was used for study quality assessment, which included three domains such as defining of study groups, between-group comparability, and validation of the outcome. This scale totally scores from 1 to 9 stars, with 9 stars indicating the highest study quality level.

### 2.4. Statistical Methods

Odds ratios (ORs) and 95% CIs were selected as the general outcome variables for the relationship between PAE and depression in offspring. Data of ORs and standard errors (SEs) were calculated from 95% CIs or P values, and an additional logarithmical transformation was performed to stabilize variance and normalize to the distribution [26, 28]. Cochrane's *Q* test was used to evaluate the heterogeneity, and the *I*^2^ statistic was also estimated [29]. Heterogeneity was deemed to be significant if *I*^2^ > 50%. A fixed-effect model was used to pool the results if the between-study heterogeneity was not significant; otherwise, a random-effect model was applied [26]. Sensitivity analyses by excluding one dataset at a time were used to evaluate the stability of the findings. Subgroup analysis was performed to evaluate the association between PAE and depression in offspring according to the study characteristics, such as design, timing for PAE evaluation, degree of PAE, average age of children/adolescents at the diagnosis of depression, adjustment of maternal smoking and maternal depression, and quality scores of the included studies. For continuous variables, medians were selected as cutoffs for defining of subgroups. According to the severity, PAE was categorized as low, moderate, and heavy conditions which was consistent with the definition of the original studies. We performed subgroup analyses according to the adjustment of maternal smoking and maternal depression because both these covariates [30, 31] have been identified as risk factors for depression in offspring. For studies reporting the dose of alcohol consumption during pregnancy (drinks per week), a univariate metaregression analysis between alcohol dose and logarithmical transformed OR (InOR) of depression was performed to evaluate potential dose-response relationship. The funnel plots were constructed, and a visual inspection of the symmetry was conducted to reflect the publication bias. Egger's regression asymmetry test was further performed for the evaluation of potential publication bias [32]. We used the RevMan (version 5.1; Cochrane Collaboration, Oxford, UK) software for the statistical analyses.

## 3. Results

### 3.1. Results of Database Search

**Figure 1:**
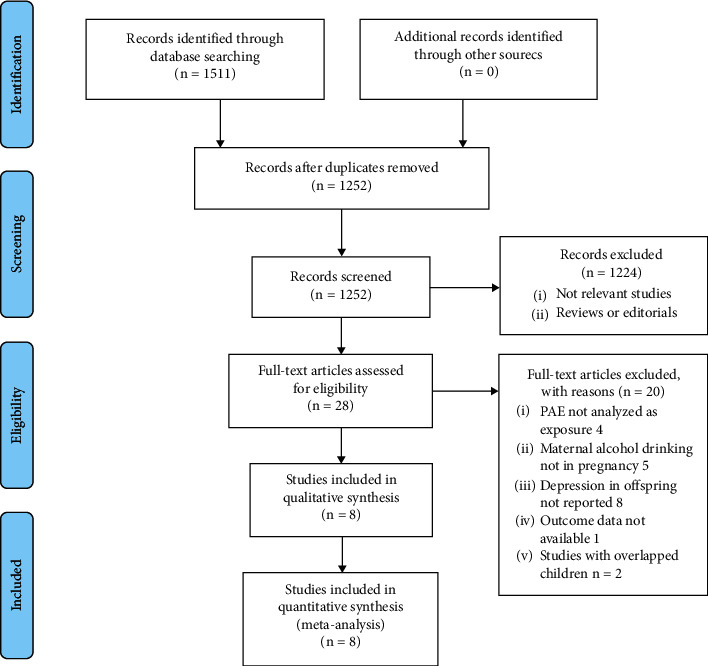
Flowchart of the database search and study identification.

The database search process is summarized in Figure 1. In brief, 1511 articles were found in the initial literature search of the Medline, Embase, and Web of Science databases; after excluding the duplications, 1252 studies remained. An additional 1224 were excluded through screening of the titles and abstracts, mainly because of their irrelevance to the meta-analysis. The remaining 28 studies underwent a full-text review. Of the 28 studies, 20 were further excluded for the reasons listed in Figure 1. Finally, eight cohort studies [16–23] were included.

### 3.2. Characteristics of the Included Studies

As shown in Table 1, eight cohort studies, including six retrospective [16–21] and two prospective cohorts [22, 23], were included. These studies were published between 2020 and 2021 and performed in the United States [16–18, 20], Australia [19, 21, 23], and the United Kingdom [22]. Overall, 7984 children and adolescents were included. The status of PAE was mostly self-reported by the mothers, while one of the included studies also applied other modalities to validate PAE, such as confirmation via medical history, birth records, social services records, and maternal report [20]. The evaluation of PAE was performed during pregnancy in two studies [22, 23], within one year after delivery in two studies [17, 19], and more than one year after delivery in the other four studies [16, 18, 20, 21]. Comparisons according to the quantitative assessment of maternal alcohol consumption in pregnancy in each study are shown in Table 1. The diagnosis of offspring depression was performed with various instruments, including the Schedule for Affective Disorders and Schizophrenia for School-Aged Children (K-SADS) [16, 18], the Pictorial Depression Scale (PDS) [17], the Child Behavior Checklist (CBCL) [19–21], the Clinical Interview Schedule-Revised (CIS-R) [22], and the Beck Depression Inventory for Youth (BDI-Y) [23]. The average age at the diagnosis of depression in the offspring varied between 5 and 18 years. Variables including demographic information of children and their mothers, social economic status, birth information, maternal smoking, and maternal depression were also adjusted to a different degree among the included studies. The NOS of the included studies ranged between six and nine stars, suggesting moderate to good quality (Table 2).

### 3.3. Meta-Analysis Results

**Figure 2:**
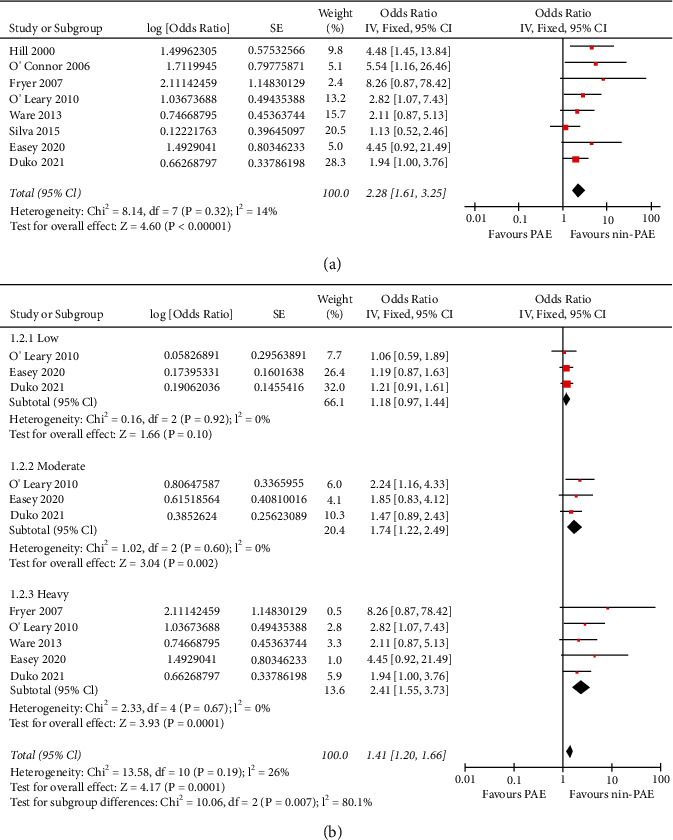
Forest plots for the meta-analysis of the association between PAE and depression in offspring: (a) results of the main meta-analysis, and (b) results of stratified analysis according to the degree of PAE.

**Figure 3:**
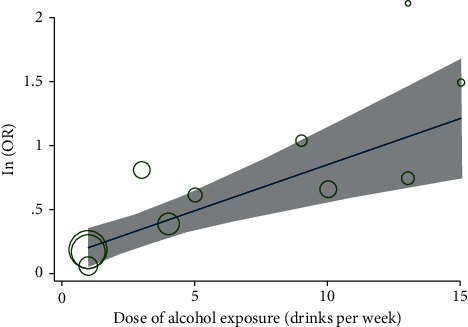
The univariate metaregression analysis between alcohol dose and logarithmical transformed OR (InOR) of depression shows a potential dose-response relationship between PAE and offspring depression.

Eight cohort studies were available for the meta-analysis and the heterogeneity among the included studies was not significant (*p* for Cochrane's *Q* test = 0.32, *I*^2^ = 14%). Pooled results with a fixed-effect model showed that PAE was associated with a higher risk of depression in offspring (OR: 2.28, 95% CI: 1.61 to 3.25, *p* < 0.001; Figure 2(a)). Sensitivity analyses by excluding one study at a time did not significantly change the results (OR: 2.12∼2.74, *p* all <0.05). Stratified analysis showed that moderate (OR: 1.74, 95% CI: 1.22 to 2.49, *p*=0.002, *I*^2^ = 0%) or heavy (OR: 1.41, 95% CI: 1.55 to 3.73, *p* < 0.001, *I*^2^ = 0%) maternal alcohol drinking in pregnancy was associated with depression in the offspring, but not for those with low maternal alcohol drinking (OR: 1.18, 95% CI: 0.97 to 1.44, *p*=0.10, *I*^2^ = 0%; Figure 2(b)). For five studies that reported the dose of PAE as drinks per week [18–20, 22, 23], univariate metaregression analysis showed a dose-response association between PAE and offspring depression (coefficient 0.073, 95% CI: 0.019 to 0.127, *p*=0.014; Figure 3). Further subgroup analyses showed that the association between PAE and risk of depression in offspring was not significantly affected by the design of the study, timing of PAE evaluation, average age of the offspring at the diagnosis of depression, adjustment of maternal smoking or depression, and differences in quality scores (*p* < 0.05 for each subgroup; Table 3).

### 3.4. Publication Bias

**Figure 4:**
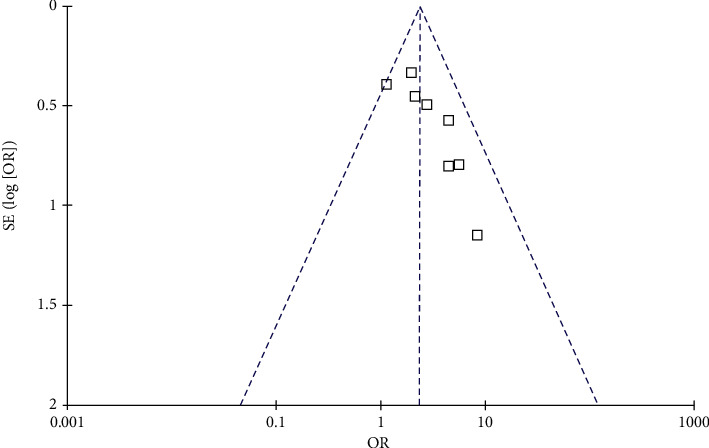
Funnel plots for the publication bias underlying the meta-analysis of the association between PAE and depression in offspring.

Figure 4 shows the funnel plots regarding the meta-analysis of the relationship between PAE and depression in offspring. Visual inspection found symmetry of the plots, which suggested a low risk of publication bias. Results of Egger's regression tests also suggested a low risk of publication bias (*p*=0.16).

## 4. Discussion

In this meta-analysis, by combining the results of available cohort studies, we found that PAE was associated with a higher risk of depression in offspring. These results suggested that maternal alcohol exposure during pregnancy may be related with the incidence of depression in offspring, particularly for those with moderate to heavy maternal alcohol consumption. Accordingly, these results highlighted the possible importance of screening and prevention of PAE for reducing the incidence of depression in offspring.

To the best of our knowledge, this is the first meta-analysis regarding the association between PAE and the risk of depression in offspring. The strengths of the methodology included extensive literature searching, including cohort studies with multivariate analyses, and applying comprehensive sensitivity and subgroup analyses to validate the findings. All the included studies were multivariate-adjusted cohort studies, which could therefore provide a possible independent association between PAE and subsequent risk of depression in offspring. Sensitivity analysis by excluding one study at a time showed that the results of the overall meta-analysis were not primarily driven by either of the included studies, which further confirmed the robustness of the findings. Although only mild heterogeneity was detected among the included studies (*I*^2^ = 14%), subgroup analyses were also performed to evaluate the possible influences of study characteristics on the outcome. These findings further confirmed that the association between PAE and a higher risk of offspring depression was not significantly affected by various study characteristics. Interestingly, we also found that the association between PAE and offspring depression was significant for moderate to heavy maternal alcohol consumption but not for low maternal alcohol consumption. In addition, a possible dose-response relationship between PAE and the risk of depression in offspring was also suggested in the meta-regression analysis. Although these findings may suggest a dose-dependent association between PAE and offspring depression, these results should be interpreted with caution because the limited datasets included and the definition for the different extent of PAE was not always consistent in the included studies.

The possible mechanisms underlying the association between PAE and offspring depression remain not fully determined. Early preclinical studies in rats showed that PAE could lead to the fetal reprogramming of HPA and gonadal systems and subsequently enhanced the susceptibility of offspring to depression/anxiety-like disorders [33, 34]. Later studies revealed that changes of glutamatergic and *γ-*aminobutyric acid (GABA) neurotransmissions were altered after PAE in offspring, which may also be involved in the pathogenesis of depression [35]. Recent studies showed that PAE reshaped the cocaine- and amphetamine-regulated transcript peptide [36] and the brain-derived neurotrophic factor of the brain [37, 38], both of which also participated in the development of depression in offspring. More studies are warranted to determine the major molecular pathways underlying the association between PAE and offspring depression and to identify the possible interventional opportunities involved.

Our meta-analysis also has limitations. Firstly, the included studies were from three countries, and it is not known if the association between PAE and depression in offspring remains in studies of other countries, such as those of Asia or Africa. Studies are warranted for further investigation. Secondly, maternal alcohol consumption during pregnancy was self-reported in almost all of the included studies. The reliability of the data regarding the exposure may affect the outcome of the meta-analysis. In addition, the diagnostic instruments for depression varied among the included studies, which may also lead to between-study heterogeneity. Moreover, the possible dose-response relationship between PAE and the risk of depression in offspring should be validated in large-scale prospective cohort studies because of the limited datasets included.

In conclusion, results of the meta-analysis showed that PAE is associated with a higher risk of depression in offspring, particularly for those with moderate to heavy maternal alcohol consumption. Accordingly, screening and prevention of PAE may potentially be effective for reducing the incidence of depression in offspring.

## Figures and Tables

**Table 1:** Characteristics of the included cohort studies.

Study	Country	Design	Number of children	Timing of PAE assessment	Validation of PAE	Comparisons	Depression diagnostic methods	No. of offspring with depression	Average age of children at diagnosis	Variables adjusted/matched
Hill 2000 [16]	USA	RC	150	8–18 years after delivery	Self-reported	Moderate-heavy versus abstinent-light PAE	K-SADS	NR	11	Maternal age, maternal smoking, SES, and parental ASPD

O'Connor 2006 [17]	USA	RC	42	1 year after delivery	Self-reported	Moderate-heavy versus abstinent-light PAE	PDS	10	5	Maternal age

Fryer 2007 [18]	USA	RC	69	About 10 years after delivery	Self-reported	Heavy versus no PAE	K-SADS	7	12	Age and sex of children and SES

O'Leary 2010 [19]	Australia	RC	1327	3 months after delivery	Self-reported	Heavy versus no PAE in first trimester	CBCL	35	8	Maternal age, ethnicity, parity, marital status, SES, smoking and illicit drug use during pregnancy, and postnatal anxiety and depression

Ware 2013 [20]	USA	RC	344	8–16 years after delivery	Self-reported	Heavy versus no PAE	CBCL	21	13	Maternal age, ethnicity, and sex of children

Silva 2015 [21]	Australia	RC	321	6–16 years after delivery	Self-reported	PAE versus no PAE	CBCL	68	13	Maternal age, child age, gender, SES, prematurity, low birth weight, parent anxiety, and depression

Easey 2020 [22]	UK	PC	4563	Within pregnancy	Self-reported	Heavy versus no PAE	CIS-R	NR	18	Maternal age, SES, homeownership, marital status, maternal education, gender, parity, maternal smoking, maternal illicit drug use, maternal depression, and partner alcohol use

Duko 2021 [23]	Australia	PC	1168	Within pregnancy	Self-reported	Heavy versus no PAE	BDI-Y	272	17	Maternal age, SES, marital status, ethnicity (race), parity, planned pregnancy, sex of child, history of maternal psychiatric disorder, maternal depression and smoking, preterm birth, birth weight, and paternal smoking status during pregnancy.

USA, United States of America; UK, United Kingdom; RC, retrospective cohort; PC, prospective cohort; PAE, perinatal alcohol exposure; K-SADS, Schedule for Affective Disorders and Schizophrenia for School-Aged Children; PDS, Pictorial Depression Scale; CBCL, Child Behavior Checklist; CIS-R, Clinical Interview Schedule-Revised; BDI-Y, the Beck Depression Inventory for Youth; NR, not reported; SES, socioeconomic status; ASPD; parental antisocial personality disorder.

**Table 2:** Quality of the included studies via the Newcastle-Ottawa Scale.

Cohort study	Representativeness of the exposed cohort	Selection of the nonexposed cohort	Ascertainment of exposure	Outcome not present at baseline	Control for age	Control for other confounding factors	Assessment of outcome	Enough long follow-up duration	Adequacy of follow-up of cohorts	Total
Hill 2000 [16]	0	1	0	1	1	1	1	1	1	7
O'Connor 2006 [17]	1	1	0	1	1	0	1	0	1	6
Fryer 2007 [18]	0	1	0	1	1	1	1	1	1	7
O'Leary 2010 [19]	1	1	1	1	1	1	1	0	1	8
Ware 2013 [20]	0	1	1	1	1	0	1	1	1	7
Silva 2015 [21]	0	1	0	1	1	1	1	1	1	7
Easey 2020 [22]	1	1	1	1	1	1	1	1	1	9
Duko 2021 [23]	1	1	1	1	1	1	1	1	1	9

**Table 3:** Results of subgroup analyses.

Study characteristics	Datasets number	OR (95% CI)	*I* ^2^ (%)	*p* for subgroup effect	*p* for subgroup difference
Study design					
Retrospective	6	2.33 [1.51, 3.58]	31	<0.001	
Prospective	2	2.20 [1.19, 4.05]	0	0.01	0.88

Timing of PAE evaluation					
Within 1 year after delivery	4	2.57 [1.57, 4.19]	0	<0.001	
More than 1 year after delivery	4	2.02 [1.22, 3.35]	42	0.007	0.50

Average age at depression diagnosis					
Within 12 years	4	3.99 [2.11, 7.55]	0	<0.001	
More than 12 years	4	1.79 [1.17, 2.73]	0	0.007	0.04

Adjustment of maternal smoking					
Yes	4	2.64 [1.65, 4.22]	0	<0.001	
No	4	1.90 [1.12, 3.24]	42	0.02	0.37

Adjustment of maternal depression					
Yes	4	1.88 [1.22, 2.89]	14	0.004	
No	4	3.39 [1.83, 6.25]	0	<0.001	0.12

Quality score					
6–7	5	2.22 [1.37, 3.60]	43	0.001	
8–9	3	2.36 [1.41, 3.95]	0	0.001	0.87

OR, odds ratio; CI, confidence interval; PAE, prenatal alcohol exposure.

## Data Availability

The data used to support the findings of this study are available from the corresponding author upon request.
